# Sleep-disordered breathing and lung function abnormalities in adults with congenital heart disease

**DOI:** 10.1007/s11325-023-02899-w

**Published:** 2023-08-08

**Authors:** D. Momcilovic, B. Reznakova, F. Bosse, C. Begrich, C. Bernhardt, M. Hamiko, F. Bakhtiary, G. Nickenig, D. Skowasch, Carmen Pizarro

**Affiliations:** 1 Department of Internal Medicine II - Cardiology, Pneumology, Angiology, University Hospital Bonn, Venusberg-Campus 1, 53127 Bonn, Germany; 2 Department of Pediatric Cardiology, University Hospital Bonn, Bonn, Germany; 3 Department of Cardiac Surgery, University Hospital Bonn, Bonn, Germany

**Keywords:** Sleep-disordered breathing, Polygraphy, Lung function abnormalities, Adults with congenital heart disease

## Abstract

**Purpose:**

Advances in treatment enables most patients with congenital heart diseases (CHD) to survive into adulthood, implying the need to address comorbid conditions in this growing cohort of patients. The aim of this study was to evaluate the prevalence of sleep-disordered breathing (SDB) and lung function abnormalities in patients with adult congenital heart disease (ACHD).

**Methods:**

Patients with ACHD underwent level 3 sleep testing (Embletta MPR polygraphy) and pulmonary function testing. Results were stratified by the underlying haemodynamic ACHD lesion group.

**Results:**

Patients with ACHD (n = 100) were middle-aged (42.3 ± 14.6 years), 54% male and slightly overweight (BMI 25.9 ± 5.5 kg/m^2^). Polygraphy revealed a prevalence of sleep apnoea of 39% with 15% of patients presenting with predominantly obstructive apnoeic episodes, while 23% of patients presenting primarily with central sleep apnoea. The distribution of mild, moderate, and severe sleep apnoea in the total study population was 26%, 7% and 6%, respectively. Comparison of apnoea–hypopnoea index, presence of sleep apnoea, and apnoea severity did not offer significant differences between the four ACHD lesion groups (*p* = 0.29, *p* = 0.41 and *p* = 0.18, respectively). Pulmonary function testing revealed obstructive lung disease in 19 of 100 patients. Concomitant chronic obstructive pulmonary disease and obstructive sleep apnoea were diagnosed in 3% of patients and were associated with profound nocturnal desaturation.

**Conclusion:**

The findings suggest a mild propensity amongst patients with ACHD to develop SDB that seems to be unaffected by the specific underlying congenital lesion.

## Introduction

Due to advances in diagnosis and treatment, survival to adulthood of patients with congenital heart diseases (CHD) has improved over the last decades. Nonetheless, little is known about pulmonary comorbidities that may be a modifiable disorder in this vulnerable population. Their presence is often underdiagnosed, as their common cardinal symptoms of dyspnoea and fatigue are frequently falsely attributed to the known cardiac condition. Within the spectrum of pulmonary comorbidities, sleep-disordered breathing (SDB) exerts an independent negative effect on cardiovascular function in the general population [1]. Obstructive sleep apnoea (OSA) is characterized by upper airway collapsibility that leads to repetitive apnoeas [2, 3]. The resulting cycles of hypoxaemia might be particularly detrimental in adult CHD (ACHD). Fluid retention and leg oedema have been reported to be accompanied by a nocturnal fluid shift from the legs to the neck and peripharyngeal soft tissues, increasing the propensity to pharyngeal narrowing in heart failure patients [4]. This mechanistic approach supports the notion that adult patients with CHD may be especially susceptible to SDB. Once diagnosed, OSA treatment in ACHD is challenging as positive pressure ventilation impedes systemic venous return by the elevation in intrathoracic pressure and may consequently have deleterious haemodynamic consequences if applied indiscriminately.

Apart from SDB, CHD influences the respiratory system through multiple physiological and anatomical mechanisms. Lung function abnormalities may arise from pulmonary congestion that decreases lung compliance [5]. Fluid retention can even affect the bronchial walls with resultant wheezing. Of note, the co-occurrence of obstructive airflow limitation and OSA, known as “overlap syndrome”, constitutes a distinct disease entity that is characterized by profound nocturnal hypoxaemia and deranged gas exchange during daytime [6, 7]. These effects might be deleterious to the ACHD cohort.

In keeping with this, the aim of the present study was to investigate prospectively the occurrence of SDB in ACHD as a function of the underlying specific CHD lesion and to correlate the results with those obtained by pulmonary function assessment.

## Methods

### Patient population

For this prospective study, consecutive patients aged ≥ 18 years were enrolled between April 2018 and December 2019. All patients received outpatient treatment at the Department of Cardiology, Hospital of Bonn (Bonn, Germany) for ACHD at the time of study inclusion, none were locally hospitalized. Exclusion criteria comprised previously diagnosed SDB or lung function abnormalities, as well as hospitalization due to CHD within 6 months prior to entry into the study. For the assessment of comorbidities, smoking habits and concomitant medication, a questionnaire-based clinical evaluation was employed and medical reports were appraised. All patients underwent overnight screening for SDB, pulmonary function testing and 6-min walk test (6-MWT). The study was approved by the local ethic committee of the faculty of medicine of the University of Bonn (Germany) and conducted in accordance with the Declaration of Helsinki. Informed written consent was obtained from all patients.

In conformity with the current guidelines on ACHD management [8], patients were subdivided according to the underlying specific lesion into four groups, namely (a) shunt lesions, (b) left-sided obstructive lesions, (c) right-sided lesions and (d) complex lesions.

### Nocturnal SDB screening

All participants underwent an overnight in-home polygraphy by a validated level 3 Embletta MPR PG device (Natus Inc., Middleton, USA) that recorded chest and abdominal wall movements, nasal airflow, snoring, oxygen saturation, heart rate and body position. Patients were prepared at the local sleep laboratory. Episodes of disordered breathing were categorized into apnoeas and hypopnoeas. Apnoeas were defined by a complete cessation of respiratory flow or a > 90% reduction in respiratory flow from baseline for at least 10 s. Hypopnoeas were diagnosed in case of a more than 50% limitation of respiratory airflow for at least 10 s, accompanied by an oxygen saturation decrease of ≥ 3%. Events were considered obstructive, when airflow limitation was accompanied by thoraco-abdominal wall movement. Otherwise, they were classified as central.

The apnoea–hypopnoea index (AHI) was defined as the number of episodes of apnoeas and hypopnoeas per hour of sleep. According to the current manual on scoring respiratory events in sleep [9], the AHI was categorized into non-pathological (< 5/h), mild (5 to < 15/h), moderate (15 to < 30/h) and severe sleep apnoea (≥ 30/h). Events were manually scored and examined by an experienced sleep laboratory specialist. To assess subjective daytime sleepiness, all study participants completed the German version of the Epworth Sleepiness Scale questionnaire (ESS) [10, 11]. An ESS score > 9 implied excessive daytime sleepiness.

In addition to the AHI, the oxygen desaturation index (ODI) was recorded, defined as the number of oxygen saturation reductions ≥ 4% from baseline for at least 10 s/h of sleep. Moreover, nocturnal snoring, arousals and heart rate were examined.

### Pulmonary function testing, capillary blood gas analysis and 6-min walk test

Pulmonary function testing was performed in line with the European Respiratory Society guidelines [12]. Testing comprised spirometry, body plethysmography and determination of diffusion capacity for carbon monoxide by single-breath method. Parameters were assessed in absolute terms and in percentages of the predicted values, calculated automatically based on age, sex and height by a dedicated software (Bodyplethismograph Jaeger©, Alveo-Diffusionstest Jaeger©, Wuppertal, Germany). Static and dynamic measurements encompassed forced expiratory volume in 1 s (FEV_1_), forced vital capacity (FVC), Tiffeneau-index (FEV_1_/VC), total lung capacity (TLC), residual volume (RV), airway resistance (R_tot_) and diffusion capacity for carbon monoxide (DL_CO_). In case of an obstructive defect, a bronchodilator test was added to evaluate reversibility of airway constriction. Bronchodilator responsiveness was defined by an increase in FEV_1_ of at least 12% after inhalation of a short acting beta-agonist (salbutamol) [13].

Capillary blood gas analysis was performed by sample collection from the hyperaemic earlobe for evaluation of oxygenation and ventilation status. Measurements comprised partial pressure of oxygen (pO_2_), partial pressure of carbon dioxide (pCO_2_) and capillary oxygen saturation (SO_2_). Capillary blood gas analysis was performed in rest and after exercise (6-MWT).

Exercise capacity was assessed by 6-MWT, performed to ATS standards [14].

### Statistical analysis

Baseline characteristics are presented as mean ± standard deviation or median and range for continuous variables and absolute numbers (percentages) for categorical variables. Baseline characteristics, results obtained by overnight polygraphy, results obtained by pulmonary function testing and capillary blood gas analysis were compared across ACHD lesion groups. Differences in categorical variables were analysed by Fisher’s exact test. Comparisons of continuous variables were carried out by univariate ANOVA or the Kruskal–Wallis *H* test (if normality assumption was violated). Normality assumption was assessed by the use of the Kolmogorov–Smirnov test. Homogeneity of variance was assessed by the use of Levene’s test. If the global test was significant, post hoc Student’s *t* tests were applied for pairwise comparisons. Results of post hoc tests were adjusted by the Bonferroni correction. Correlation between continuous variables was calculated with Pearson’s correlation. Two-tailed *p*-values were computed and considered significant if ranging below 0.05. SPSS Statistics version 26.0 (IBM, Armonk, NY, USA) was used for all statistical analyses and graphics.

## Results

### Clinical characteristics

A total of 100 patients with ACHD were included in the study. Patients were categorized into four lesion groups according to their haemodynamics. Shunt lesions and left-sided obstructive lesions were present in 25 patients each 27 patients offered right-sided lesions, and 23 patients presented complex lesions. The specific defects are given in Table 1. The most frequent lesions comprised tetralogy of Fallot (*n* = 16), transposition of the great arteries (*n* = 13) and congenital aortic stenosis (*n* = 12). Demographic data and clinical features of study participants are displayed in Table 2. Patients were generally middle-aged (42.3 ± 14.6 years), male (54%) and slightly overweight (body mass index 25.9 ± 5.5 kg/m^2^). The vast majority of patients were never smokers (69%), whilst continued nicotine consumption was exhibited by 26% of patients. With regard to established cardiovascular risk factors, no significant differences were detected by comparison of the four lesion groups. As to cardiovascular medication use, pulmonary arterial hypertension treatment was more frequent amongst complex lesion patients (*p* = 0.02); the remaining cardiovascular medication use did not differ between groups. In terms of exercise response, physical performance was comparable within the different lesion groups, as assessed by 6-MWT distance (*p* = 0.39). Nonetheless, patients presented differences in NYHA function capacity, with higher NYHA function classes being more common in complex lesion patients (*p* = 0.01).

**Table 1:** Overview of specific underlying lesions

	All patients (*n* = 100)
Shunt lesions (*n* = 25)	
Atrial septal defect	10
Ventricular septal defect	10
Atrioventricular septal defect	3
Patent ductus arteriosus	2
Left-sided obstructive lesions (*n* = 25)	
Congenital aortic stenosis	12
Coarctation of aorta	10
Congenital mitral stenosis	2
Others	1
Right-sided lesions (*n* = 27)	
Tetralogy of Fallot	16
Pulmonary stenosis	6
Ebstein anomaly	5
Complex lesions (*n* = 23)	
Transposition of the great arteries	13
Eisenmenger syndrome	4
Coronary anomalies	2
Fontan palliation	2
Others	2

Data are presented as total number

**Table 2:** Baseline characteristics of total study population and stratified by CHD lesion groups

	All patients (*n* = 100)	Shunt lesions (*n* = 25)	Left-sided obstructive lesions (*n* = 25)	Right-sided lesions (*n* = 27)	Complex lesions (*n* = 23)	*p*-value*
Demographics						
Female	46 (46%)	15 (60%)	9 (36%)	11 (41%)	11 (48%)	0.36^C^
Age (years)	42.3 ± 14.6	43.6 ± 14.5	42.0 ± 15.8	44.7 ± 15.4	38.4 ± 12.5	0.47^a^
BMI (kg/m^2^)	25.9 ± 5.5	26.7 ± 6.5	26.5 ± 5.3	24.7 ± 3.6	25.9 ± 6.4	0.52^a^
Smoking history						
Packyears	4.3 ± 8.5	5.8 ± 10.2	4.2 ± 7.5	2.9 ± 5.7	5.6 ± 10.2	0.76^b^
Current smoker	26 (26%)	6 (24%)	8 (32%)	7 (26%)	5 (22%)	0.21^C^
Ex-smoker	5 (5%)	3 (12%)	2 (8%)	0 (0%)	0 (0%)	
Never-smoker	69 (69%)	16 (64%)	15 (60%)	20 (74%)	18 (78%)	
Cardiovascular risk factors						
Arterial hypertension	20 (20%)	7 (28%)	5 (20%)	5 (195%)	3 (13%)	0.68^C^
Diabetes mellitus	3 (3%)	1 (4%)	1 (4%)	1 (4%)	0 (0%)	1.00^C^
Dyslipidaemia	15 (15%)	5 (20%)	2 (8%)	4 (15%)	4 (17%)	0.68^C^
Obesity	18 (18%)	7 (28%)	5 (20%)	1 (4%)	5 (22%)	0.29^C^
NYHA functional class						< 0.05^c^
I	50 (50%)	14 (56%)	15 (60%)	15 (56%)	6 (26%)	
II	37 (37%)	9 (36%)	8 (32%)	8 (30%)	12 (52%)	
III	13 (13%)	2 (8%)	2 (8%)	4 (15%)	5 (22%)	
6-min walk test						
Walk distance (m)	464.1 ± 102.1	493.4 ± 92.2	466.9 ± 91.3	443.1 ± 106.3	452.5 ± 118.9	0.39^a^
Cardiovascular medication use						
Oral anticoagulant	34 (34%)	8 (32%)	8 (32%)	10 (37%)	8 (35%)	0.41^C^
Platelet inhibitor	16 (16%)	2 (8%)	6 (24%)	4 (15%)	4 (17%)	0.33^C^
RAAS-inhibitor	31 (31%)	3 (12%)	13 (52%)	5 (19%)	10 (44%)	0.07^C^
ß-Blocker	50 (50%)	10 (40%)	15 (60%)	14 (52%)	11 (48%)	0.39^C^
Statin	12 (12%)	3 (12%)	1 (4%)	5 (19%)	3 (13%)	0.32^C^
Calcium antagonist	6 (6%)	0 (0%)	1 (4.0%)	1 (4%)	1 (4%)	0.56^C^
Digitalis	4 (4%)	0 (0%)	1 (4%)	3 (11%)	0 (0%)	0.40^C^
PAH medication	5 (5%)	0 (0%)	0 (0%)	2 (7%)	3 (13%)	< 0.05^C^

Data are presented as total number and percentage (in parentheses) or mean ± standard deviation

*BMI* body mass index, *NYHA* New York Heart Association, *PAH* pulmonary arterial hypertension

^*^p-values refer to data comparison between all four CHD subgroups

^a^One-way ANOVA

^b^Kruskal–Wallis test

^c^Fisher’s exact test

### Somnological examination

**Fig. 1:**
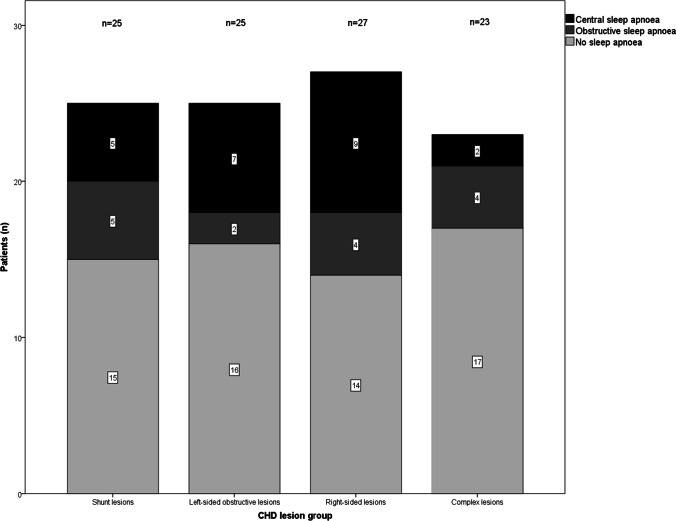
Distribution of obstructive and central sleep apnoea over CHD lesion groups

**Fig. 2:**
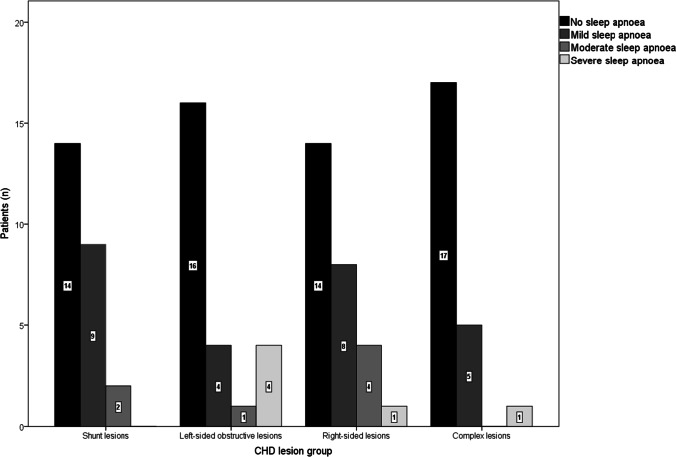
Sleep apnoea severity classes stratified by CHD lesion groups. Abbreviations: CHD: congenital heart disease

SDB results are summarized in Table 3. Within the entire study population, polygraphic screening revealed a total prevalence of sleep apnoea of 39%, with a mean AHI of 7.3 ± 11.9/h. When stratified by the obstructive or central nature of respiratory events, 15% of patients presented with predominantly obstructive apnoeic episodes, whilst 23% of patients had primarily central sleep apnoea (Fig. 1). The distribution of mild, moderate, and severe sleep apnoea in the total study population was 26%, 7% and 6%, respectively. OSA was significantly related to BMI (*p* < 0.001), age (*p* < 0.01) and gender (*p* < 0.05). Figure 2 displays AHI severity classes over lesion groups. Comparison of AHI, presence of sleep apnoea and apnoea severity between the four lesion cohorts did not offer significant differences (*p* = 0.29, *p* = 0.41 and *p* = 0.18, respectively).

**Table 3:** Results obtained by overnight polygraphy in the total study population and stratified by CHD lesion groups

	All patients (*n* = 100)	Shunt lesions (*n* = 25)	Left-sided obstructive lesions (*n* = 25)	Right-sided lesions (*n* = 27)	Complex lesions (*n* = 23)	*p*-value*
Mean recording time (min)	400.1 ± 77.6	393.0 ± 87.2	408.6 ± 83.4	397.1 ± 74.9	402.1 ± 66.3	0.91
AHI (1/h)	7.3 ± 11.9	6.2 ± 6.3	10.9 ± 20.1	7.4 ± 8.2	4.5 ± 6.9	0.29
ODI (1/h)	8.3 ± 12.6	7.8 ± 8.2	11.7 ± 20.8	8.4 ± 9.1	5.5 ± 7.3	0.40
Nocturnal oxygen saturation						
Minimal (%)	84.5 ± 8.4	83.5 ± 10.4	87.2 ± 7.5	84.2 ± 6.7	83.1 ± 8.4	0.32
Mean (%)	92.9 ± 3.9	93.5 ± 2.9	94.5 ± 1.8	92.7 ± 3.6	91.1 ± 5.7	0.13
Total snoring events	5.5 ± 17.8	9.8 ± 29.0	2.0 ± 5.0	7.7 ± 17.5	2.0 ± 6.8	0.30
Autonomic arousal (1/h)	7.2 (0–62.6)	8.1 (0–45.9)	18.5 (0–61.3)	0.8 (0–62.6)	8.5 (0–59.9)	0.38^a^
Nocturnal heart rate (beats/min)	67.5 ± 14.9	70.6 ± 18.8	69.3 ± 12.3	64.6 ± 15.8	65.5 ± 11.2	0.45
ESS	6.1 ± 4.2	4.8 ± 3.1	4.6 ± 2.1	7.8 ± 4.9	7.2 ± 5.0	0.13

Data are presented as mean ± standard deviation or median (range)

*AHI* apnoea–hypopnoea index, *ESS* Epworth Sleepiness Scale, *ODI* oxygen desaturation index

^*^p-values refer to data comparison between all four CHD subgroups by one-way ANOVA, if not stated otherwise

^a^Kruskal-Wallis test

With regard to nocturnal oxygenation status, neither the minimum nor the mean levels of oxygen saturation recorded substantially varied by intercohortal comparison (average minimum oxygen saturation ranged between 83.1% and 87.2% across groups, *p* = 0.32; average mean oxygen saturation ranged between 91.1% and 94.5% across groups, *p* = 0.13). The same holds true for the ODI (*p* = 0.40). A mean ESS of 6.1 ± 4.2 was indicative of no relevant daytime sleepiness and upheld over all four lesion groups studied (*p* = 0.13). Amongst patients with ACHD and OSA, mean ESS was 6.9 ± 4.6 and did not significantly differ neither from the ESS obtained in patients with central sleep apnoea (6.9 ± 4.1, *p* = 0.99) nor from that in patients with ACHD but no sleep-disordered breathing (4.9 ± 2.4, *p* = 0.24).

### Pulmonary function testing

As given in Table 4, pulmonary function testing revealed an overall FEV_1_/FVC ratio of 87.2 ± 10.1%. FEV_1_ accounted for 2.8 ± 0.9 l in absolute terms and 79.5 ± 17.2% of the predicted value. Obstructive lung disease was present in 19 of 100 patients in the total study population. The bronchodilator test evidenced responsiveness in 4 of 19 patients, whereas the remaining 15 patients showed bronchodilator non-response and thus were diagnosed with chronic obstructive pulmonary disease (COPD). Within the four ACHD cohorts, obstructive ventilatory defects were observed in 5%, 5%, 5% and 4% of patients with shunt lesions, left-sided obstructive lesions, right-sided lesions and complex lesions, respectively (*p* = 0.69 for intercohortal comparison). Concomitant COPD and OSA—referred to as “overlap syndrome”—were detected in 3 of 100 patients. Presence of overlap syndrome was significantly associated with nocturnal desaturation, as measured by minimum overnight oxygen saturation levels (67.5 ± 12.0% versus 84.2 ± 8.2%, *p* < 0.01).

**Table 4:** Results obtained by pulmonary function testing and capillary blood gas analysis in the total study population and stratified by CHD lesion groups

	All patients (*n* = 100)	Shunt lesions (*n* = 25)	Left-sided obstructive lesions (*n* = 25)	Right-sided lesions (*n* = 27)	Complex lesions (*n* = 23)	*p*-value*
Spirometry and body plethysmography						
TLC (L)	5.5 ± 1.5	5.8 ± 1.4	6.3 ± 1.8	4.9 ± 1.3	5.1 ± 1.2	< 0.01
TLC (% predicted)	91.4 ± 19.2	99.0 ± 22.1	97.8 ± 15.5	82.3 ± 14.8	86.8 ± 19.1	< 0.01
FVC (L)	3.4 ± 1.1	3.4 ± 1.1	3.9 ± 1.1	3.1 ± 1.1	3.2 ± 1.0	0.06
FVC (% predicted)	81.9 ± 17.2	83.9 ± 20.6	88.5 ± 12.1	77.6 ± 17.2	77.6 ± 16.2	0.09
FEV_1_ (L)	2.8 ± 0.9	2.8 ± 0.9	3.2 ± 0.8	2.6 ± 0.9	2.7 ± 0.8	0.09
FEV_1_ (% predicted)	79.5 ± 17.2	80.4 ± 21.7	86.8 ± 9.9	77.9 ± 18.4	72.6 ± 13.7	0.05
FEV_1_/VC (%)	87.2 ± 10.1	84.5 ± 8.8	87.8 ± 10.0	89.8 ± 12.3	86.3 ± 8.6	0.32
RV (L)	2.1 ± 1.0	2.4 ± 1.2	2.3 ± 1.3	1.8 ± 0.6	1.9 ± 0.8	0.08
RV (% predicted)	114.3 ± 50.9	132.5 ± 65.1	119.5 ± 46.9	96.5 ± 32.2	109.3 ± 50.4	0.09
R_tot_ (% predicted)	119.1 ± 63.0	111.2 ± 41.8	94.5 ± 21.2	143.9 ± 92.8	124.4 ± 61.1	0.05
Diffusion capacity for carbon monoxide						
DL_CO_ (% predicted)	66.6 ± 15.7	75.8 ± 19.4	68.3 ± 13.5	63.8 ± 11.2	57.8 ± 13.3	< 0.01
DL_CO_/VA (% predicted)	84.9 ± 17.8	93.0 ± 19.8	81.9 ± 14.8	86.3 ± 15.0	77.4 ± 19.2	0.04
Capillary blood gas analysis at rest						
pO_2_ (mmHg)	78.5 ± 16.9	81.7 ± 23.5	81.5 ± 6.2	77.1 ± 9.6	73.1 ± 22.3	0.37
pCO_2_ (mmHg)	35.4 ± 7.2	33.9 ± 5.5	38.8 ± 12.2	35.8 ± 3.8	32.7 ± 2.9	0.05
sO_2_ (%)	95.1 ± 6.1	93.3 ± 11.0	96.8 ± 0.7	95.8 ± 1.3	94.5 ± 3.9	0.32
Capillary blood gas analysis after exercise (6-MWT)						
pO_2_ (mmHg)	81.8 ± 16.6	82.6 ± 22.6	89.3 ± 9.0	80.3 ± 12.5	74.9 ± 18.2	0.11
pCO_2_ (mmHg)	34.8 ± 4.7	34.2 ± 5.9	34.9 ± 5.9	36.1 ± 3.9	33.6 ± 2.5	0.45
sO_2_ (%)	95.6 ± 4.5	95.5 ± 6.1	97.4 ± 0.8	95.4 ± 2.4	93.9 ± 6.5	0.27

Data are presented as mean ± standard deviation

*DL*_*CO*_ diffusion capacity of the lung for carbon monoxide, *FEV*_*1*_ forced expiratory volume in 1 s, *FVC* forced vital capacity, *pCO*_*2*_ carbon dioxide tension *pO*_*2*_ oxygen tension, *R*_*tot*_ airway resistance, *RV* residual volume, *sO*_*2*_ oxygen saturation, *VA* alveolar volume, *6-MWT* 6-min walk test

^*^p-values refer to data comparison between all four CHD subgroups by one-way ANOVA

Restrictive ventilatory disorders were diagnosed in 23 of 100 patients and significantly varied over ACHD groups. Whereas only 12% and 8% of patients with shunt lesions and left-sided obstructive lesions, respectively, presented restrictive defects, the percentage rose to 41% in the right-sided lesion group and 30% in complex lesion patients (*p* < 0.05 for intercohortal comparison). Post hoc tests showed that this observation was primarily driven by differences between left- and right-sided lesion patients (*p* < 0.05). As given in Table 4, TLC significantly varied over ACHD groups, both in absolute and relative terms (*p* < 0.01 and *p* < 0.01, respectively). The Bonferroni adjustment ascribed this effect to differences between the right-sided lesion group, on the one hand, and the shunt (*p* < 0.05) and left-sided obstructive lesion group (*p* < 0.05), on the other hand (values given for TLC absolute). The same holds true for results obtained for diffusion capacity that decreased over ACHD groups and was lowest in complex lesion patients. DL_CO_ positively correlated with TLC (Pearson’s *r* = 0.38, *p* < 0.001 for TLC absolute and Pearson’s *r* = 0.44, *p* < 0.001 for TLC of the predicted value). Both DL_co_ and TLC were related to exercise capacity in terms of 6-MWT distance (Pearson’s *r* = 0.43, *p* < 0.001 for TLC absolute and Pearson’s *r* = 0.37, *p* = 0.001 for DL_CO_ of the predicted value).

## Discussion

The present study prospectively analysed the prevalence of sleep apnoea and lung function abnormalities in an ACHD study population. The main findings are as follows: (i) Sleep apnoea was diagnosed in 39% of patients with ACHD and was predominantly central in nature. (ii) Neither prevalence nor severity of sleep apnoea substantially differed over ACHD lesion groups. (iii) ACHD was accompanied by a remarkable proportion of ventilatory defects of both obstructive and restrictive nature, of which restrictive defects related to the underlying congenital lesion.

Due to remarkable gains in survival, the field of ACHD is witnessing rapid growth. It has led to a shift in the demographics of CHD, where adults now outnumber children by a ratio of 2:1 [15]. In this growing cohort of patients, comorbid conditions—both related to the underlying defect and acquired—are gaining attention, such that improvements in understanding can affect lifelong care. Evidence has emerged to support a substantial prevalence of sleep apnoea in ACHD. However, the currently assessed proportion of patients with concomitant sleep apnoea only slightly exceeded its estimated prevalence in the general, middle-aged population of 5–25% [16]. Prior studies have principally focused on single defects. In a cohort of 22 adult patients who had previously underwent Fontan palliation of single ventricle physiology, polysomnography was retrospectively analysed and revealed an SDB frequency of 77% [17]. This prevalence notably exceeds the one ascertained in our study. This discrepancy might primarily be driven by screening bias, as the aforementioned trial retrospectively examined more symptomatic patients who had undergone polysomnography in routine clinical care and thus had a higher pre-test probability of SDB. Miles and colleagues performed in-home overnight oximetry in patients with CHD and pulmonary valve dysfunction and detected decreased nocturnal oxygen saturation levels in 13 of 22 patients (59%) [18]. Within our group of right-sided lesions, SDB prevalence indeed rose to 48% but was still well below the one reported by Miles et al. This inconsistency might be ascribed to less diagnostic accuracy arising from only oximetry. In a cohort of 20 patients with Eisenmenger syndrome, OSA was polysomnographically diagnosed in 15% of patients, which is equal to the OSA prevalence found in our ACDH study population [19]. Consistent with our findings, OSA did not correlate with the underlying type of shunt but with BMI and age. Drake et al. examined a broader spectrum of CHD lesions by the use of the Berlin Questionnaire that stratifies patients into risk classes of having OSA [20]. They identified a proportion of 31% of patients with ACHD at high risk of OSA that diverges from our results, mainly due to the employed screening tool. A likewise wider range of defects was evaluated by Harada and colleagues [21]. Patients with ACHD who required hospitalization and underwent in-hospital overnight polygraphy were retrospectively analysed. A total of 63% of patients were diagnosed with sleep apnoea with the broad majority obstructive in nature. These data are opposed to our findings and surpass our SDB frequency by far. Diverging results might be traced back to a different study population. Harada et al. examined patients hospitalized for ACHD, presumably implying cardiac decompensation, whilst our study participants were outpatient and excluded from study entry in case of hospitalization due to CHD within the preceding 6 months. In acquired left heart failure, central sleep apnoea is primarily driven by fluid excess in the pulmonary interstitium with elevated pulmonary wedge pressure that stimulates juxtapulmonary receptors and affects chemoresponsiveness of the respiratory centre [22]. However, the mechanisms underlying central abnormalities of SDB in ACHD are less well defined. In infants with cardiomyopathy, central sleep apnoea was observed in 24% of patients [23]. Of note, severity of central sleep apnoea (central apnoea–hypopnoea index) correlated with the left ventricular end diastolic volume index. It supports the concept that left heart failure—whether congenital or acquired—is accompanied by pulmonary congestion that contributes to the severity of sleep apnoea [4].

In terms of lung function abnormalities in ACHD, prior studies have primarily focused on restrictive pulmonary defects. The underlying mechanisms leading to restrictive lung function in ACHD are diverse. They comprehend a less favourable foetal environment and lower physical activity in childhood that compromise lung development. Pulmonary congestion and respiratory muscle weakness, in particular diaphragm dysfunction, may additionally cause restrictive ventilatory defects, the latter being reported to be accompanied by increased inflammation levels [24]. Alonso-Gonzalez and colleagues analysed spirometry data in 1188 ACHD patients and reported a prevalence of 47% of markedly abnormal FVC [25]. The presence and severity of the restrictive defect were related to the complexity of the underlying cardiac lesion. This observation is consistent with our findings insomuch as TLC significantly varied over ACHD groups (Table 4). However, our pulmonary function testing was not limited to spirometry but additionally included body plethysmography to inform on lung volumes and diffusion capacity testing to provide a window on mechanistic inference. DL_CO_ positively correlated with TLC, supporting the notion of an underlying causative pulmonary congestion. Both TLC and DL_CO_ were related to exercise capacity.

With regard to obstructive pulmonary defects, Singh et al. studied non-cardiac comorbidities in hospitalized patients with ACHD in a US database and detected COPD in 20% of patients [26]. This proportion slightly outnumbers the frequency of COPD assessed in our study (15%). Noteworthy, we identified obstructive lung disorders in 19% of patients but complemented bronchodilator testing to differentiate between COPD and asthma. Neither obstructive lung defects in general nor COPD differed in frequency over the four ACHD groups. In the present study, we ascertained a prevalence of 3% of overlapping OSA with COPD. This result is consistent with the observations made in the Sleep Heart Health Study by Sanders et al.: the prevalence of OSA was not greater in patients with versus without COPD and ranges from 5 to 25% [27]. In light of our findings, patients with ACHD do not appear to be prone to the development of overlapping OSA and COPD. Like in the general unselected population, the concomitance of both disorders was associated with profound nocturnal desaturation [7].

A study limitation arises from the participation of  a single hospital, a fact that impeded the enrollment of a larger number of patients with ACHD. Study protocol did not allot a control population that would have permitted direct comparison of occurrence of SDB and lung function disorders in healthy controls. Furthermore, we used ambulatory polygraphy for SDB screening, whilst the “gold standard” is overnight attended polysomnography. Though level 3 portable devices have been attributed a high degree of diagnostic accuracy [28], polysomnography would have been a valuable adjunct to validate polygraphy results.

To summarize, patients with ACHD exhibited only a slightly higher vulnerability to sleep apnoea than that reported for the general population. The findings suggest a mild propensity amongst patients with ACHD to develop SDB that is related to established risk factors such as gender, age, and obesity but seems to be unaffected by the underlying congenital lesion. ACHD was accompanied by ventilatory defects of both obstructive and restrictive nature. 

## Data Availability

The datasets generated and/or analysed during the current study are available from the corresponding author on reasonable request.
